# Contractile behavior of the gastrocnemius medialis muscle during running in simulated hypogravity

**DOI:** 10.1038/s41526-021-00155-7

**Published:** 2021-08-09

**Authors:** Charlotte Richter, Bjoern Braunstein, Benjamin Staeudle, Julia Attias, Alexander Suess, Tobias Weber, Katya N. Mileva, Joern Rittweger, David A. Green, Kirsten Albracht

**Affiliations:** 1 Department of Medical Engineering and Technomathematics, University of Applied Sciences Aachen, Aachen, Germany; 2 German Sport University Cologne, Institute of Movement and Neurosciences, Cologne, Germany; 3 German Sport University Cologne, Institute of Biomechanics and Orthopaedics, Cologne, Germany; 4 Centre for Health and Integrative Physiology in Space (CHIPS), Cologne, Germany; 5 German Research Centre of Elite Sport, Cologne, Germany; 6 King’s College London, Centre of Human and Applied Physiological Sciences, London, UK; 7 European Astronaut Centre (EAC), European Space Agency, Space Medicine Team (HRE-OM), Cologne, Germany; 8 KBR GmbH, Cologne, Germany; 9 London South Bank University, School of Applied Sciences, London, UK; 10 Institute of Aerospace Medicine, German Aerospace Center (DLR), Cologne, Germany; 11 Department of Pediatrics and Adolescent Medicine, University of Cologne, Cologne, Germany; 12 Institute for Bioengineering, University of Applied Sciences Aachen, Aachen, Germany

**Keywords:** Ultrasonography, Outcomes research, Physiology

## Abstract

Vigorous exercise countermeasures in microgravity can largely attenuate muscular degeneration, albeit the extent of applied loading is key for the extent of muscle wasting. Running on the International Space Station is usually performed with maximum loads of 70% body weight (0.7 g). However, it has not been investigated how the reduced musculoskeletal loading affects muscle and series elastic element dynamics, and thereby force and power generation. Therefore, this study examined the effects of running on the vertical treadmill facility, a ground-based analog, at simulated 0.7 g on gastrocnemius medialis contractile behavior. The results reveal that fascicle−series elastic element behavior differs between simulated hypogravity and 1 g running. Whilst shorter peak series elastic element lengths at simulated 0.7 g appear to be the result of lower muscular and gravitational forces acting on it, increased fascicle lengths and decreased velocities could not be anticipated, but may inform the development of optimized running training in hypogravity. However, whether the alterations in contractile behavior precipitate musculoskeletal degeneration warrants further study.

## Introduction

Astronauts exposed to a micro-g-force environment, often referred to as microgravity (μg), experience many physiological adaptations, including musculoskeletal deconditioning, with the plantar flexor muscles appearing particularly susceptible to atrophy^1,2^. To prevent these detrimental effects, the crewmembers of the International Space Station (ISS) perform daily exercise countermeasures, including treadmill running, cycling, and resistance training^3^. Due to the implementation of new exercise hardware and improvements of the in-flight exercise hardware and exercise prescriptions, μg-induced physiological deconditioning has been reduced, although variable inter-individual physiological responses to the exercise induced stimuli persist^4,5^.

For instance, a recent study investigating the plantar flexor muscles of two ISS crewmembers suggests that vigorous treadmill and resistive training reduces the decrements in muscle volume and lower limb strength and the deteriorations in muscle architecture^6^. Moreover, the muscle wasting seems to affect the organism’s systemic inflammatory/anti-inflammatory balance^7^, which highlights the requirement to safeguard musculoskeletal health in space. The extent of muscle wasting is likely related to the training volume (sets, repetition, and duration) and training intensity in particular with regard to the maximum external loading that can comfortably be applied during countermeasure exercises^6^.

ISS crewmembers that are part of U.S. Orbital Segment currently perform locomotion countermeasures on the T2 treadmill. On this treadmill, subject loading is currently provided via a harness system connected to a bungee assembly that is clipped in series with several carabiner clips. The applied harness load is usually an individual crew choice, mainly limited by increasing discomfort of the harness system at higher loads (A. Gerst, Personal Communication 2021, see Supplementary Reference). Running sessions are thus usually performed with ~70% of the equivalent body weight (BW) at 1g (*g* = 9.81 m s^−2^)^3^, resulting in lower peak ground reaction forces (~1.3 BW when running at 2.2 m s^−1^) compared to terrestrial running^8^. A ground-based analog to simulate hypogravity running on the ISS is the vertical treadmill facility (VTF), where subjects are suspended horizontally with graded “pull-down” forces toward a vertically mounted treadmill provided via a harness-based subject loading system^9–11^. Despite marginal differences in joint kinematics and ground reaction forces between running in actual μg (parabolic flight) vs. running in simulated μg (VTF), the latter is still regarded as a valid analog, even though it does not provide a 1:1 representation of running in actual µg since on the VTF the (vertically suspended) body and in particular the suspended limbs are still exposed to gravity^12^.

Running on the ISS or on ground-based hypogravity simulation systems is not only associated with reduced ground reaction forces but also with lower plantar load^13–15^. In addition, metabolic cost was found to be reduced during running in simulated hypogravity^15–17^. Furthermore, estimated ankle joint forces^18^ and peak ankle dorsiflexion and knee flexion as well as range of motion^19^ were reported to be reduced when running at different velocities (2.2‒3.5 m s^−1^) on a lower body positive pressure treadmill to simulate hypogravity.

In contrast, running with additional mass (120% of BW, equivalent to 1.2 g), was found to require more mechanical work at the ankle and knee joints^20^. Despite these changes in kinetic gait parameters, the overall gastrocnemius medialis (GM) muscle fascicle behavior and peak series elastic element (SEE) length were found to be largely preserved. Interestingly, essentially preserved fascicle−SEE behavior was also observed when walking with only 70% BW achieved by lower body positive pressure^21^. In 1 g, whilst changes in walking speed between 0.75 and 2.00 m s^−1^ have been shown to affect fascicle velocity (at the time of peak force), no effects were observed when changing running speed between 2.00 and 3.25 m s^−1^ ^22^. Taken together, these findings make it difficult to predict if and how the neuromuscular system modulates fascicle‒SEE dynamics when running in simulated hypogravity.

When reducing the loading level, gait transitions occur at a slower preferred walk-to-run transition speed (PTS) but at a similar Froude number, a dimensionless number embedding gait speed, leg lengths, and gravitational acceleration^23–25^. Thus, to run at “dynamically similar” speeds (i.e., at a similar running speed relative to the PTS) in hypogravity, one must run at the same Froude number, which means a reduction in absolute running speed. However, the influence of hypogravity running at a dynamically similar speed on the interaction between the contractile and series elastic elements within GM’s muscle‒tendon unit (MTU) has not been investigated.

Therefore, the aim of this study was to investigate, via ultrasonography, GM fascicle−SEE behavior in addition to joint kinematics during running with 125% of the PTS at a simulated hypogravity level of 0.7 g (on the VTF) versus 1 g. We hypothesized that fascicle−SEE behavior will be preserved when running in simulated hypogravity at 125% of the PTS.

## Results

### Kinetic and spatio-temporal parameters

**Fig. 1:**
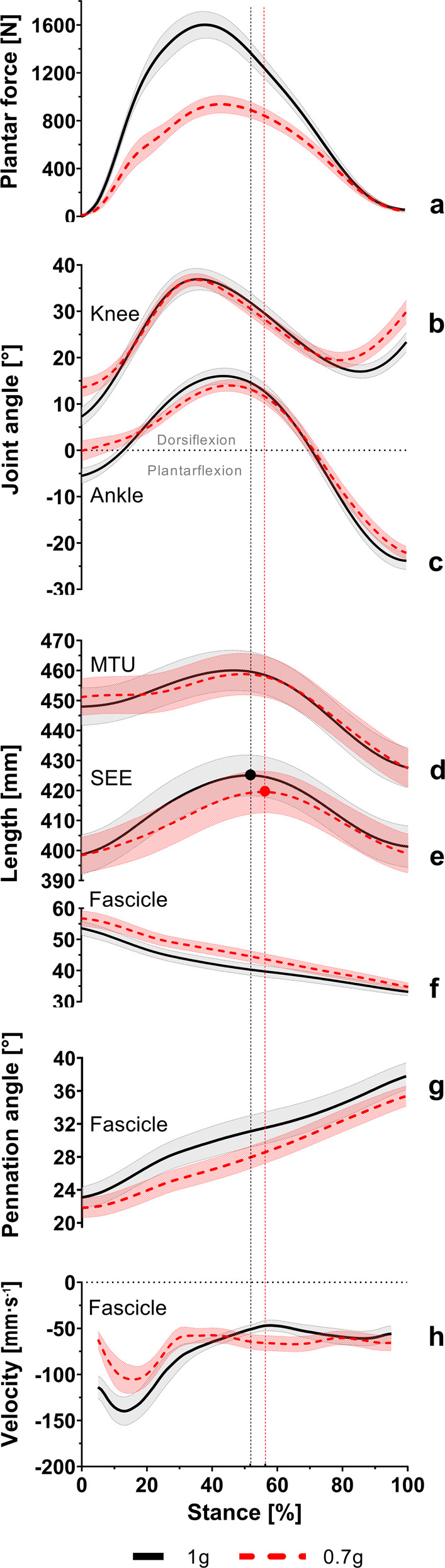
Kinetic, kinematic, and GM fascicle‒SEE parameters during the stance phase of running at 1 g and simulated 0.7 g. Participants’ average (mean ± standard error of the mean) patterns of plantar forces (**a**), knee (**b**) and ankle (**c**) joint angles, and MTU (**d**) and SEE (**e**) lengths as well as muscle fascicle length (**f**), pennation angle (**g**), and velocity (**h**) change during the stance phase of running at 1 g (black line) and simulated 0.7 g (red dashed line). The vertical lines mark the peak SEE length achieved at 1 g (black) and simulated 0.7 g (red). *n* = 8 participants.

Running speeds in this study were selected to correspond to 125% of the participants’ PTS, resulting in average running speeds of 2.62 ± 0.08 m s^−1^ at 1 g and 2.23 ± 0.07 m s^−1^ at simulated 0.7 g. Participants running on the VTF at simulated hypogravity of 0.7 g were subjected to lower (*t*(7) = 11.465, *P* < 0.001, *d*_z_ = −4.1) mean loading levels than at 1 g, corresponding to 63.4 ± 4.8% (mean ± standard deviation) of the loading levels determined during running on a conventional treadmill. Peak plantar forces (*t*(7) = 9.070, *P* < 0.001, *d*_z_ = −3.2) were reduced by 633.3 ± 197.5 N (95% confidence interval (CI), −798.4 to −468.2) at simulated 0.7 g compared to 1 g (Fig. 1a). In contrast, ground-contact times (*t*(7) = 5.597, *P* < 0.001, *d*_z_ = 2.0) were increased by 0.05 ± 0.02 s (95% CI, 0.03 to 0.07) when running at simulated 0.7 g (Table 1). Accordingly, cadence (*t*(7) = 5.442, *P* = 0.001, *d*_z_ = −1.9) was decreased by 10.1 ± 5.2 steps min^−1^ (95% CI, −14.4 to −5.7) at simulated 0.7 g compared to 1g.

**Table 1:** Spatio-temporal and kinematic parameters while participants ran at 125% of their preferred walk-to-run transition speed at 1 g and simulated 0.7 g.

Parameters	1 g	0.7 g	*P* value
Ground-contact time [s]	0.30 ± 0.04	0.35 ± 0.04^a^	<0.001
Cadence [steps min^−1^]	83.25 ± 5.90	73.19 ± 4.21^a^	0.001
Ankle joint range of motion [°]	40.18 ± 7.72	36.44 ± 5.99	0.155
Knee joint range of motion [°]	30.03 ± 5.15	25.69 ± 3.65^a^	0.018
Ankle dorsiflexion [°]	21.91 ± 3.92	15.11 ± 5.02^a^	<0.001
Knee flexion [°]	29.98 ± 5.20	24.06 ± 4.99^a^	0.003
Ankle joint angle at peak SEE length [°]	15.19 ± 5.09	12.81 ± 4.06^a^	0.016
Knee joint angle at peak SEE length [°]	31.92 ± 6.25	28.63 ± 4.75^a^	0.030

Data are presented as mean ± standard deviation.

^a^significantly different (two-tailed paired *t*-test) from 1 g (*P* ≤ 0.05). Peak SEE length at simulated 0.7 g and 1 g occurred at 57 ± 4% and 52 ± 7% of stance, respectively. *n* = 8 participants

### Joint kinematics

The participant’s knee and ankle joint movement patterns during running at simulated 0.7 g vs. 1 g are displayed in Fig. 1b,c, respectively.

Knee joint range of motion (*t*(7) = 3.057, *P* = 0.018, *d*_z_ = −1.1) was lower by 4.3 ± 4.0° (95% CI, −7.7 to −1.0) at simulated 0.7 g compared to 1 g, whereas ankle joint range of motion (*t*(7) = 1.595, *P* = 0.155, *d*_z_ = −0.6) was not affected by unloading, with a mean difference of 3.7 ± 6.6° (95% CI, −9.3 to 1.8) (Table 1). Furthermore, ankle dorsiflexion (*t*(7) = 6.629, *P* < 0.001, *d*_z_ = −2.3) and knee flexion (*t*(7) = 4.503, *P* = 0.003, *d*_z_ = −1.6) during the first half of the stance phase were both lower at simulated 0.7 g, by 6.8 ± 2.9° (95% CI, −9.2 to −4.4) and 5.9 ± 3.7° (95% CI, −9.0 to −2.8), respectively (Table 1). At the time of peak SEE length, both ankle (*t*(7) = 3.144, *P* = 0.016, *d*_z_ = −1.1) and knee (*t*(7) = 2.706, *P* = 0.030, *d*_z_ = −1.0) joint angles were lower during running at simulated 0.7 g, by 3.0 ± 2.7° (95% CI, −5.3 to −0.7) and 3.3 ± 3.4° (95% CI, −6.2 to −0.4), respectively (Table 1).

### GM muscle and SEE parameters

Temporal differences in muscle−SEE parameters within the single stance phase between running at simulated 0.7 g and 1 g are depicted in Fig. 1d−h.

**Fig. 2:**
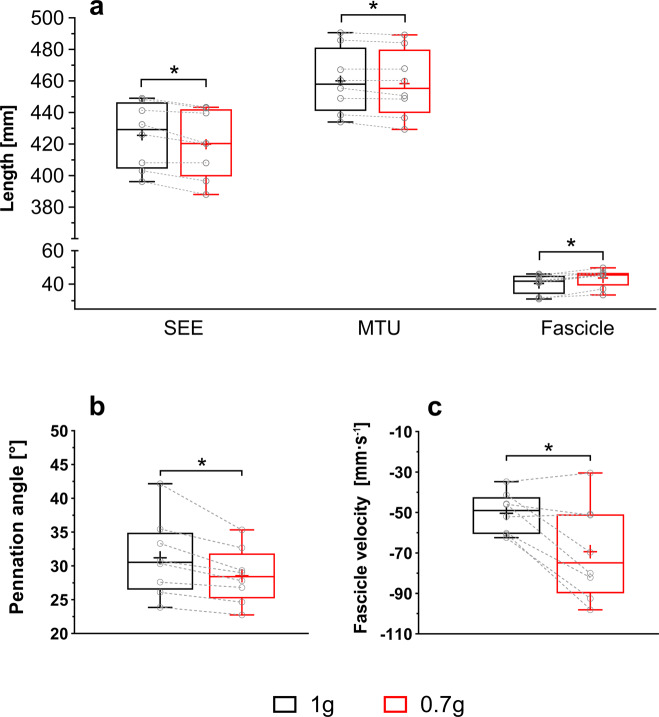
GM fascicle‒SEE behavior at the time of peak SEE length when running at 1 g and simulated 0.7 g. SEE length (**a**, left), MTU length (**a**, middle), fascicle length (**a**, right), pennation angle (**b**), and fascicle velocity (**c**) at the time of the peak SEE length differ between running at 1 g (black box) and simulated 0.7 g (red box). The lower and upper parts of the box represent the first and third quartile, respectively. The length of the whisker represents the minimum and maximum values. The horizontal line in the box represents the statistical median of the sample; + the mean of the sample; ○ individual data points; * significantly different (two-tailed paired *t*-test) from 1 g (*P* ≤ 0.05). *n* = 8 participants.

Loading level had no effect upon overall fascicle shortening (*t*(7) = 1.646, *P* = 0.144, *d*_z_ = 0.6), with a mean difference of 1.6 ± 2.7 mm (95% CI, −0.7 to 3.9). However, at the time of peak SEE length, muscle fascicles operated at a longer length (∆ = 3.3 ± 1.9 mm, 95% CI 1.7 to 4.9, *t*(7) = 4.922, *P* = 0.002, *d*_z_ = 1.7, Fig. 2a), smaller pennation angle (∆ = −2.7 ± 2.0°, 95% CI −4.3 to −1.0, *t*(7) = 3.789, *P* = 0.007, *d*_z_ = −1.3, Fig. 2b), and faster shortening velocity (∆ = 19.0 ± 16.6 mm s^−1^, 95% CI −32.9 to −5.1, *t*(7) = 3.230, *P* = 0.014, *d*_z_ = −1.1, Fig. 2c) at simulated 0.7 g compared to 1 g.

The peak SEE length (*t*(7) = 4.315, *P* = 0.004, *d*_z_ = −1.5) and the MTU length at the time of peak SEE length (*t*(7) = 2.547, *P* = 0.038, *d*_z_ = −0.9) were shorter during running at simulated 0.7 g compared to 1 g, by 5.6 ± 3.7 mm (95% CI, −8.7 to −2.5) and 1.8 ± 2.0 mm (95% CI, −3.5 to −0.1), respectively (Fig. 2a).

The time at which peak SEE length was achieved (*t*(7) = 1.860, *P* = 0.105, *d*_z_ = 0.7) did not differ between loading levels although peak SEE length was attained slightly later (56.9 ± 4.1% vs. 51.5 ± 7.5% of stance phase, 95% CI, −1.5 to 12.2) at simulated 0.7 g compared to 1 g.

## Discussion

Running on the VTF with 125% of the PTS at simulated 0.7 g vs. 1 g induced peak plantar forces corresponding to ~1.3 BW, which are similar to those observed when running with bungee loading on board the ISS^8^. The main findings of the present study were that simulated 0.7 g running increased ground-contact time, reduced cadence, and lowered ankle dorsiflexion and knee flexion at the time of peak SEE length. Concurrently, GM fascicles operated at longer lengths, smaller pennation angles, and faster shortening velocities, whilst MTU and SEE lengths were shorter.

Hypogravity (0.7 g) running induced a significant reduction in peak plantar forces (−39.3%), whilst ground-contact times were slightly increased, resulting in a greater time available for the neuromuscular system to adopt GM’s contractile behavior. In addition, ankle and knee joint angles at the time of the peak SEE length were significantly reduced, consistent with a previous lower body positive pressure study reporting that participants adapted their running pattern when the loading level was <0.8 g^19^. Smaller ankle joint flexions were also observed in studies investigating running on a treadmill equipped with a vertical body weight support system^26^ or a subject loading system as used during parabolic flights^10^. Changes in GM’s MTU behavior may thus result from an altered movement pattern induced by prolonged stance phase durations and altered joint kinematics. For instance, lower ankle dorsiflexion at peak SEE length may compensate for the less-flexed knee joint, significantly shortening MTUs.

Furthermore, the present study indicates that at the time of peak SEE length, where the force acting on the SEE is at its greatest, GM fascicles are less contracted compared to 1 g running. Simultaneously, the pennation angle was found to be significantly smaller, potentially facilitating fascicles to operate at longer lengths. However, this contrasts with preliminary findings indicating that overall fascicle behavior is relatively stable against a reduction or increase in loading by 30% BW^21^ or 20% BW^20^, respectively.

During 1 g running, fascicles barely reach the plateau region of the force−length relationship^27,28^, thereby limiting their ability to generate force. Thus, the increase in fascicle length observed during simulated (0.7 g) hypogravity running may enable the GM to operate closer to its optimum length, thereby enhancing force-generation ability. By using a normalized active force−length relationship represented by a Gaussian function^29^ and an optimum GM fascicle length of 51.0 ± 9.8 mm (99% confidence interval, 45.0–58.0 mm), as determined by a cadaveric study^30^, we estimate an increase in GM’s force-generation ability by as much as 6% when running at simulated 0.7 g. Moreover, a shift in the fascicles’ operating range toward longer lengths may result in an increased strain on the z-disks, potentially preserving or increasing the number of sarcomeres in-series, which in turn may be beneficial for muscle mass preservation^31^.

On the other hand, fascicle-shortening velocity at peak SEE length was found to be significantly increased during running at simulated 0.7 g. This may result in less-favorable contractile conditions as fascicles are less able to generate force with increasing speed of contraction^32^. In fact, it has been reported that fascicle-shortening velocity is a major determinant of the preferred walk-to-run transition by improving fascicles’ contractile conditions after switching gait to counteract impaired muscle force production^22,33^. Indeed, a change in fascicle velocity is noted to have a greater impact on muscular performance than a change in fascicle length, especially at high running speeds^34^.

Interestingly, at 1 g, fascicles operated at a sub-optimal length but at a slower and more optimal shortening velocity for generating force. Assuming that fascicle neuro-motor control is optimally adapted to 1 g, an increase in fascicle velocity and thus induction of less-favorable contractile conditions in simulated 0.7 g may outweigh the benefits from an increase in fascicle length. In fact, the significantly shorter peak SEE length observed during simulated 0.7 g running may be the direct result of lower muscular forces acting on the SEE. In addition, according to the MTU’s stretch-shortening cycle, the smaller SEE strain should result in less stored and thus released elastic energy. However, as an exact replication of running in actual microgravity is not possible using the VTF, the present results strongly suggest but do not prove that running on the ISS induces a significant change in muscle‒tendon dynamics in response to lower musculoskeletal loading.

Although, given a largely preserved fascicle behavior when running with increased loading^20^, one might speculate that when the musculoskeletal loading is increased, it is more important to preserve the well-adapted contractile conditions to favor economical force production.

It has been proposed that the provision of (non-standardized) external force loading while exercising in μg may underlie the observation of variable muscular degeneration during long-term spaceflight^6^. The present (in vivo) study supports this notion as significant alterations in GM fascicle−SEE outcome parameters were observed between running at simulated 0.7 g on a ground-based analog and 1 g. Such alterations point to functional adaptations in response to a reduced locomotor demand during hypogravity running, involving not only lower gravitational but also muscular forces that may precipitate musculoskeletal degeneration^35^. Thus, it appears that the consequences of hypogravity running are not limited to a mere reduction in mechanical loading but also to an altered contractile behavior, which could affect the muscle’s work capacity upon the return to daily activities in a 1 g environment. The longer fascicles may be beneficial to preserve muscle mass but may also result in long-term adaptations in optimal fascicle length that are no longer functional for the requirements on Earth and may require specific attention during the rehabilitation phase upon return to Earth’s 1 g environment^31,36^.

To increase the mechanical loading on the MTU during hypogravity running, and hence to induce muscle fascicle−SEE behavior that is similar to that in 1 g conditions (to ensure the stimuli exerted on the muscle remain the same), it would be required that the harness applies a higher external force to the body than what is typically chosen by crewmembers. This force is mainly limited by considerable discomfort of the harness at higher loads, especially during gait cycle phases with maximum stretch of the bungee assembly^3,37^. Moreover, whether the provision of full BW loading is actually optimal, is subject to further research. An alternative approach may be to increase running speed^8,15,38^, which has been shown to augment maximum plantar force^13^. Interestingly, many ISS crewmembers appear to intuitively increase their running speed to achieve a perceived workout intensity that is similar to what they are used to on Earth despite the reduced external loading (A. Gerst, Personal Communication 2021, see Supplementary Reference). This sensation may relate to the fact that increasing running speed reduces the ground-contact time but requires a higher force production, evident by greater plantar flexor muscle activity^20^. However, further research is needed to investigate the interaction between loading level and running speeds on fascicle−SEE behavior in vivo. Another possibility to mitigate μg-induced muscle wasting would be to increase the volume of in-flight treadmill running. However, one goal of optimizing exercise countermeasures in space is the reduction of crew time spent on exercise whilst maintaining or improving the effectiveness of currently prescribed exercise countermeasures^39^.

To conclude, simulated 0.7 g running significantly alters fascicle-SEE interaction. For instance, a shorter peak SEE length seems to be the result of lower muscular forces acting on it. However, to answer the question as to whether there is a loading and running speed combination above which muscular deconditioning is prevented, additional measurements of torque and neuromuscular activation are required to estimate the effects of (various-level) hypogravity running on GM strain and resultant contractility/excitability. Such knowledge is crucial to inform the development of optimized running training in hypogravity but may also inform the mechanisms of contractile behavior regulation on Earth.

## Data processing

For each participant and each outcome measure at each loading level, the first eight consecutive left foot stance phases (from the 30 s of data recording) were analyzed using a custom-made script (MATLAB R2018a, MathWorks, Inc., Natick, United States). Fascicle length and pennation angle data were smoothed with a five-point moving average, whereas electrogoniometer signals were smoothed with a fifth-order Butterworth low-pass filter at a 10-Hz cut-off frequency. Muscle fascicle velocities were calculated as the time derivative of the respective length using the central difference method^40^. Data were time-normalized by being resampled to 101 data points per stance phase.

To estimate the loading achieved on the VTF, average simulated gravity levels over the stance phase were calculated via plantar force and impulse and expressed as percentage of the average gravity levels determined similarly during running on a conventional treadmill. Peak plantar force was defined as the maximum force value observed during stance. Ground-contact times were calculated as the time between left foot touchdown and toe-off. Cadence was defined as steps (duration from touchdown to the next ipsilateral touchdown) per minute. Ankle and knee joint angles as well as SEE-, fascicle-, and MTU lengths in addition to fascicle pennation angle and velocity were determined at the time of the peak SEE length, where the force acting on the SEE is at its greatest. Overall fascicle shortening was calculated by subtracting the minimum from the maximum fascicle length. Ankle and knee joint ranges of motion were defined as the differences between their respective minimum and maximum joint angles. The differences in knee and ankle joint angles between touchdown to the time of first local maximum and maximum dorsiflexion were defined as knee flexion and ankle dorsiflexion, respectively.

### Statistical analysis

Data distribution for all outcome measures was assessed using the Shapiro−Wilk normality test. As normal distribution was confirmed for all outcome measures, a two-tailed paired *t*-test (*n* = 8 participants) was performed to test for significant differences in joint kinematics and fascicle‒SEE outcomes between loading levels (1 g vs. simulated 0.7 g). All statistical analysis was performed in GraphPad Prism (v 7.04) with *α* set to 0.05. Data is reported as mean (±standard deviation). Effect sizes (*d*_z_) were calculated using the G*Power software version 3.1.9.4^41^. Thresholds of 0.2, 0.5, and 0.8 were defined as small, moderate, and large effects between the two comparison groups^42^.

## Methods

### Participants

Eight healthy male volunteers (31.9 ± 4.7 years, 178.4 ± 5.7 cm heights, 94 ± 6 cm leg lengths, and 73.5 ± 7.3 kg body masses) provided informed written consent to participate in this study, which received approval from the “Ärztekammer Nordrhein” Ethical Committee of Düsseldorf, Germany, in accordance with the ethical standards of the 1964 Helsinki declaration. All participants were examined medically. Exclusion criteria included cardiovascular, musculoskeletal, or neurological diseases and/or surgery within 2 years prior to participation.

### Study design and experimental protocol

**Fig. 3:**
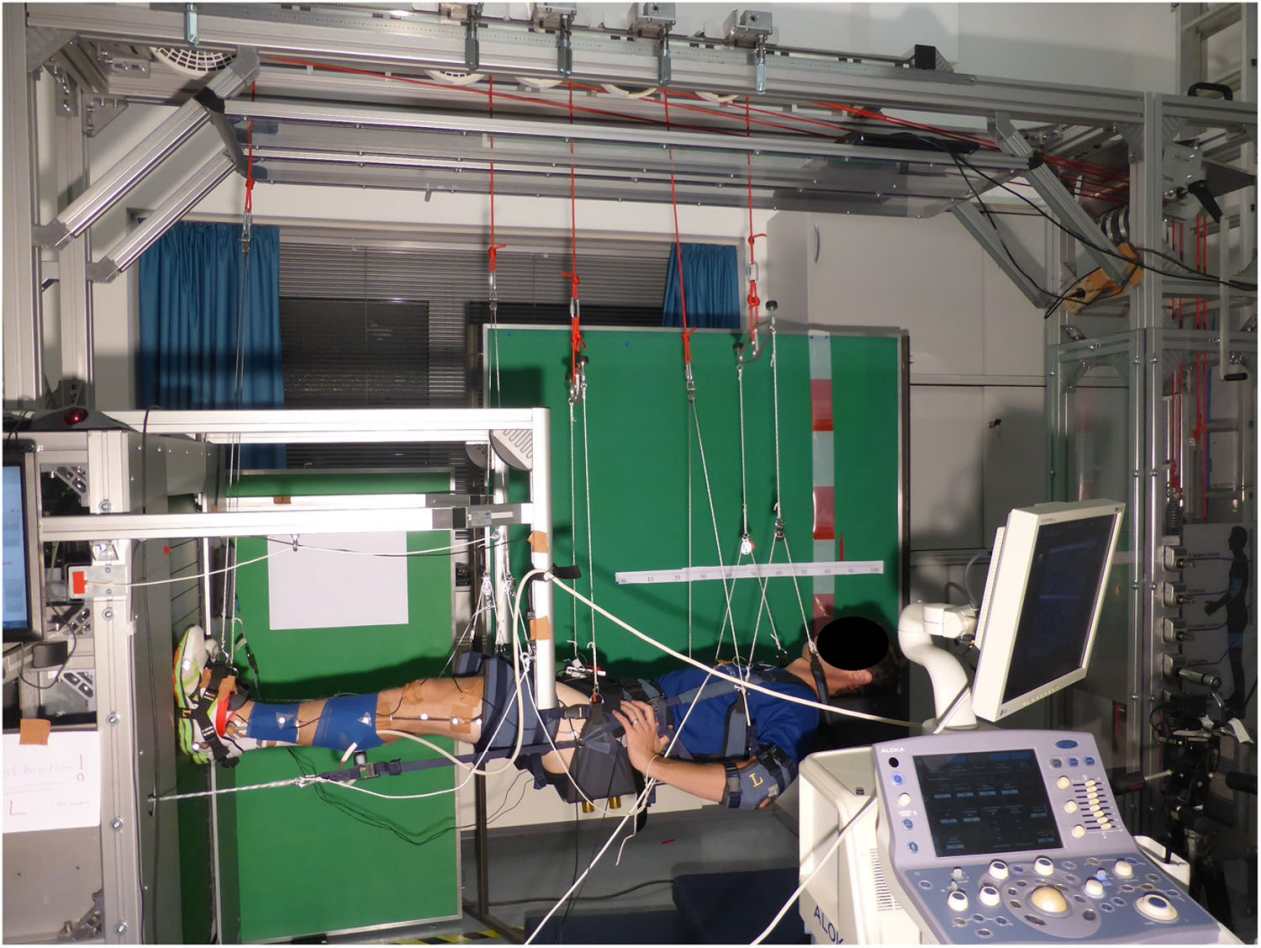
VTF experimental set-up. Participant being suspended horizontally on the vertical treadmill facility (VTF) with an ultrasound transducer attached to the mid-belly of the GM muscle and electrogoniometers to record knee and ankle joint angles. Photo credit Charlotte Richter; participant provided written informed consent to publish this photo.

Participants attended the laboratory on a single occasion and familiarized themselves with running on the vertical treadmill facility (VTF; Arsalis, Glabais, Belgium, Fig. 3) at their predefined running speed (125% PTS). After achieving a stable gait, 30 s were recorded while they ran on the VTF at simulated 0.7 g in addition to on a conventional treadmill at 1 g.

The VTF comprises a customized, motorized treadmill (Woodway, Waukesha, WI, USA) mounted vertically onto a chassis with an overhead suspension system, allowing supine suspension of the participant using a customized cradle and harness. Fabric cuffs attached to cords support the participants’ torso and pelvis and each foot, thigh, and arm. An adjustable piston-based loading system generates a constant controllable force pulling the participant toward the treadmill belt via fixation to the harness at the pelvis (Fig. 3). Written informed consent was obtained for publication of this photograph (Fig. 3).

At both loading levels, running speeds were defined as 125% of the PTS to obtain mechanically equivalent running speeds. PTS, expressed as a Froude number (PTS_FR_), was estimated by fitting an exponential regression equation $({\mathrm{PTS}}_{{\mathrm{FR}}}(a) = 1.183e^{ - 5.952a} + 0.4745)$ with a least-squares method (r^2^ = 0.99) to the data provided by Kram, et al.^23^ using the resulting acceleration (*a*) as the independent variable. Hence, for *a* = 0.7 g, a PTS_FR_ value of 0.49 was obtained. By accounting for the participants’ leg length (*l*), the individual ${\mathrm{PTS}}(a) = \sqrt {{\mathrm{PTS}}_{{\mathrm{FR}}}\left( a \right) \cdot a \cdot l}$ was determined for each participant; moreover, adding 25% to this PTS resulted in running speeds of 2.62 ± 0.08 m s^−1^ at 1 g and 2.23 ± 0.07 m s^−1^ at simulated 0.7 g.

### Data collection

#### Joint kinematics

Knee and ankle joint angles were recorded using a twin-axis (Penny and Giles Biometrics Ltd., Blackwood Gwent, UK) and a custom-made 2D-electrogoniometer, respectively. The end blocks of the knee electrogoniometer were positioned along the line from the greater trochanter to the lateral femur epicondyle and from the lateral femur epicondyle to the lateral malleolus. The end blocks of the ankle electrogoniometer were placed along the line from the lateral femur epicondyle to the lateral malleolus and from the lateral malleolus to the most distal end of the fifth metatarsal bone. Before each running trial, the goniometers were zeroed when in the anatomical neutral position (standing). Electrogoniometry data were sampled at 1500 Hz via the TeleMyo 2400 G2 Telemetry System (Noraxon USA., Inc., Scottsdale, USA) using the MyoResearch XP software (Master Edition 1.08.16). Electrogoniometry has been revealed to produce reliable and reproducible knee and ankle joint kinematics^43–45^ and has already been used during running with reduced loading^19,26,46,47^.

#### Spatio-temporal parameters

Shoe insoles (novel GmbH, loadsol^®^ version 1.4.60, Munich, Germany) were used to measure plantar forces during running and hence to determine the stance phase. Touchdown and toe-off were automatically detected from the signal acquired with a sampling rate of 83 Hz via a custom-made script (MATLAB R2018a, MathWorks, Inc., Natick, United States) using a 20 N force threshold for 0.1 s. Insole and electrogoniometer signals were time-synchronized via recording of a rectangular TTL pulse generated by pressing on a custom-made pedal before each running trial.

#### GM muscle fascicle length and pennation angle

Real-time B-mode ultrasonography (Prosound α7, ALOKA, Tokyo, Japan) was used to image the GM fascicles at a frame rate of 73 Hz. The T-shaped 6-cm linear array transducer (13 MHz), placed inside a custom-made cast to prevent shifting, was positioned at the intersection of the mediolateral and proximodistal midline over the GM mid-belly and secured with elastic Velcro. The ultrasound recordings and electrogoniometer signals were time-synchronized via a rectangular TTL pulse generated by a hand switch recorded on the electrocardiography channel of the ultrasound device and the MyoResearch XP software. Ultrasonography has been frequently used in dynamic conditions^48^ and is regarded as a reliable method to quantify fascicle architecture. Fascicle length and pennation angle show good reproducibility not only within sessions but also between sessions^49,50^.

**Fig. 4:**
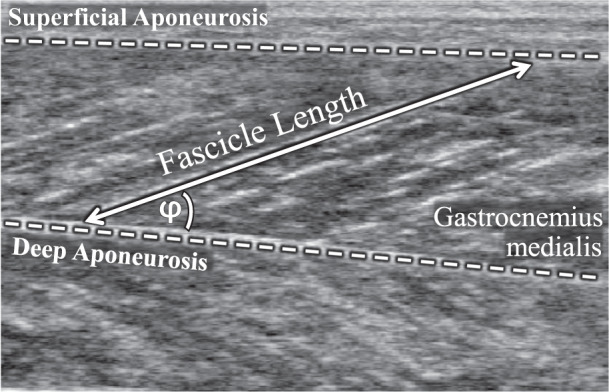
Ultrasound image of the gastrocnemius medialis with schematic representation of the extracted fascicle parameters. The pennation angle (φ) of the muscle fascicles (double-headed arrow) is defined with respect to the deep aponeurosis (lower dashed line). Fascicle length is measured as the length following the pennation between the deep and the superficial (upper dashed line) aponeuroses.

A semi-automatic tracking algorithm (UltraTrack Software, version 4.2)^51^ was used to quantify GM fascicle lengths and pennation angles. Manual correction of the digitized fascicle and the deep aponeurosis, defined as a second fascicle, was performed where appropriate. Fascicle length was defined as the distance between the insertions to the superficial and deep aponeurosis parallel to the lines of collagenous tissue (Fig. 4). If the transducer’s field of view was too small to display the entire fascicle, the missing portion was extrapolated, assuming that the fascicle and the aponeuroses extended linearly. The pennation angle (φ) was defined as the angle between the fascicle and the deep aponeurosis (Fig. 4).

#### SEE and MTU length

To calculate SEE length (Achilles tendon, aponeuroses, and proximal tendon) on the basis of an MTU model^52^, muscle fascicle lengths multiplied by the cosine of their pennation angles were subtracted from the MTU lengths. MTU length was calculated via a multiple linear regression equation^53^ using the participant’s shank length as well as their knee and ankle joint angles.

## Supplementary information

Supplementary Reference

nr-reporting-summary_NPJMGRAV-00567R4

## Data Availability

The data that support the findings of this are available from the corresponding author upon reasonable request.

## Code availability

A custom-made MATLAB code (MathWorks, Inc., Natick, United States) used for data analysis is available from the corresponding author upon reasonable request.
