# Supplementary material for: Contractile behavior of the gastrocnemius medialis muscle during running in simulated hypogravity
Source: NPJ Microgravity. 2021 Aug 9;7:32. doi: 10.1038/s41526-021-00155-7 (PMC8352871; doi:10.1038/s41526-021-00155-7)
Supplement: Supplementary file 1 — Supplementary Reference [file 41526_2021_155_MOESM1_ESM.pdf]

Supplementary Reference

From: Alexander Gerst
Sent: Monday, February 22, 2021 12:00
To: Tobias Weber
Subject: Personal Communications on ISS exercise - Feb 2021

Dear Tobias Weber, dear colleagues,

Many thanks for the interesting questions about our use of the T2 treadmill and general exercising on ISS. I hope that the information is of use for you and the scientific community, it would be a pity if that data would be left unused. Also, it is good to clarify these questions instead of making assumptions. Below I will include my own personal experience of ISS exercise. While many ISS crewmembers exercise in a very similar way, there is nevertheless a degree of personal variation, thus I would like to caution from extrapolating these statements. Please feel free to share them as official personal communications with your community and for use in publications. Additionally, please find two photos attached for explanation (photo credit A. Gerst / ESA / ISS crew).

When I flew to ISS in 2014 and 2018, I used ARED, T2 and CEVIS as exercise devices. In terms of workout protocols, we are typically free to choose. I personally used T2 as an aerobic workout opportunity that provides useful impact forces on my musculoskeletal system, but also provides me with a mental break and an experience that gets as close as possible to a "run in the forest" back on Earth. It is important to understand, and to take into account in the future, that workouts in space serve not only for physical conditioning, but also as a mental relaxation, just as on Earth. I.e., simply increasing workout intensities and reducing workout times will only address a part of the objectives of exercise on future space missions, and might even lead to a reduced overall benefit for the crewmembers if it is not addressed with the other objectives in mind.

On T2, I typically ran 3-4 times per week for 30-35 mins at ~10 km/h average, each intersected with short (1-3 mins) intervals of 12-14 km/h intervals (still in the aerobe range, i.e. not super high intensity). I have not analysed this in detail, but I feel that most crewmembers, when running on T2, intuitively select a running speed that allows them to achieve a similar "workout experience" (e.g. exertion) than what they are used from running on Earth, leading to roughly similar heart rates. That probably means that most of us increase their running speeds somewhat to counteract the reduced g-load. I never did high intensity interval training on T2, because it's not my personal preference.

For strapping and loading ourselves onto the T2 treadmill, we used two bungee assemblies that were clipped in series with several carabiner clips each, attaching to the harness fixation points on the left and right side of my hip. These clips serve to reduce the stretch on the bungee, and thus to lower the load on the harness, since the force would be too high for comfort if the bungee were used without the clip extension (see photo attached). Crewmembers typically adjust the number of clips to match their body height with the desired loading. I believe that many, if not most of the crewmembers limit this load to about 65%-70%

equivalent body weight loading. This is due to harness discomfort, despite not all clips being removed at that setup. I.e. the bungee system would typically allow for an even higher loading (even though possibly not up to 100%, depending on the body height and mass of the subject). At the end of my first mission, during which I slowly increased my loading over the course of several months by subsequently removing clips, and during most of my second mission, I had 3-4 clips left, but already achieved about 65-70% of my body weight of 90-92 kgs, at a body height of 186 cm. Using an even higher load would have felt quite uncomfortable.

Some more detail about the harness system: The T2 harness system has four major points of force application (right and left hip bones and shoulders). In contrast to 'normal' running on Earth, this results in a change of the body's centre of pressure with much more forces acting on the shoulders. Thus, running on ISS is comparable to running with a heavy backpack on Earth (not in terms of overall weight, but in terms of being "top-loaded"), which is, in addition to the high forces on those four points, the reason for the perceived discomfort (see Fig. 2). Thus, even if a higher force loading could be applied, crewmembers might still wish to stick to approximately 70% of their body weight. It would be interesting to see if the use of more sophisticated loading systems (like for example SLS) makes any difference in this dynamic, by applying more constant forces even at the top of the gait. But my feeling is that this is a general problem of being loaded through a surface force instead of a volume force like on Earth.

My other cardio training was on the CEVIS cycle ergometer, also 2-3 times per week for 30-40 mins (average 160-180 W, peaks around 250 W). I used clip-in pedals to allow for pushing and pulling of the pedals. To counteract pedal forces, I used a hip belt when exercising on CEVIS, which pulled my hip "down" and "back" (in body coordinates), and thus prevented my upper body from being pushed away from the pedals. This setup allowed me to apply stronger "downward" push forces than "upward" pull forces on the pedals, which is similar to cycling on Earth, allowing me to produce a similar power output and exertion than on Earth. Note: without the belt, I could only apply much lower loads on CEVIS, mainly dominated by the requirement to fully compensate upward and downward forces on the pedals, to a zero residual force. I assume from my own case that, typically, the muscles that are pulling the pedals are much less developed than the ones that are pushing the pedals. Accordingly, I clearly experienced that a belt-less setup on ISS results in a much less efficient use of the device in terms of overall cardio exertion, or it forces the crewmember to counter-react residual pedal forces via their hands (which is quite uncomfortable). Some crewmembers also use their T2 harness instead of a hip belt, according to their personal preference, which allows for less upper body motion, but it also works.

I exercised strongly, i.e. near max strength, on ARED 6 times per week, building up loads over the course of the mission. ARED was the key element for my ability to retain muscle mass. Additionally, during all my ARED exercise sessions, in between the individual exercise repetitions, I used the ARED hardware (various bar height settings) for stretching various muscle groups, which felt quite pleasant (in space the possibilities to stretch muscles are very rare, due to the requirement to counteract all stretch forces, which is much easier in a 1-g environment than in space).

I did not have significant muscle deconditioning when I came back from space (this, however varies from crewmember to crewmember). In fact my overall muscle mass increased during

both my missions by a few kg, mainly contributed by the large muscle groups being stimulated on ARED. There might have been small losses in smaller muscle groups though (e.g. calf muscle or spine supporters), depending on the individual level of workout of that muscle. Yet, if there were any such deconditioning effects, I couldn't feel them, and they did not impact my workouts on Earth after coming back. In fact, for several exercises like squats or bench press, after returning from space I could push more loads than before, due to the strict ARED workout regime on ISS. Some of those muscle groups also showed a noticeable increase in volume after return (mainly pectoralis & abdominis). I know of several other crewmembers who told me that they made an overall similar experience after working out consistently and strongly on ARED.

About a week after the return of my second space flight, from my personal perspective, there was not much that told me that I had been to space. I remember a few secondary effects like slightly swollen lower limbs for 2-3 weeks, but that was it. However, I would like to stress again that this is only my own personal experience, which should not be extrapolated to a wider pool of crewmembers. Despite this effect, I was careful not to overload myself after return, to give my smaller muscles, spinal discs and overall coordination time to adapt back to "Earth dynamics". Therefore, I feel that despite the abovementioned lack of significant loss of muscle mass, a several-week-long reconditioning regime was still very important and useful for me.

Most USOS (US operated segment) crewmembers follow a roughly similar training regime than what I described above, but there are still significant variations in intensity, as preferred by the individual crewmember, and thus differences in physiological response. Much larger differences in the training regimes exist between the sub-groups of USOS crewmembers and Russian Segment crewmembers, but still with a high degree of individual variation.

Kind regards,
Alexander Gerst

Figure 1 T2 running system with harness, bungee assembly and carabiner clips (with friendly permission by A. Gerst / ESA).

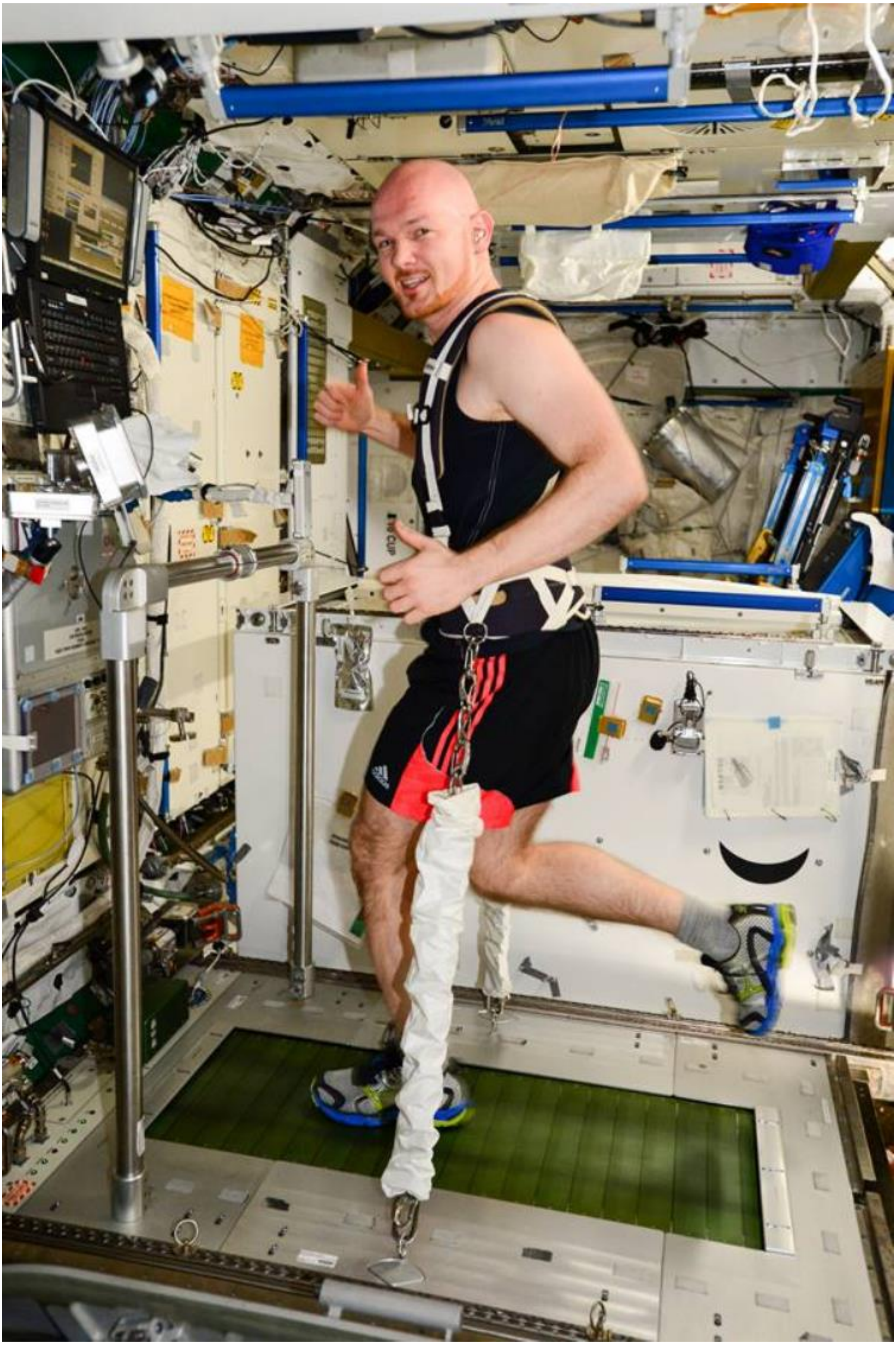

Figure 2 Typical chaffing and pressure marks on a crewmember's body, right after completing a T2 workout (with friendly permission by A. Gerst / ESA).

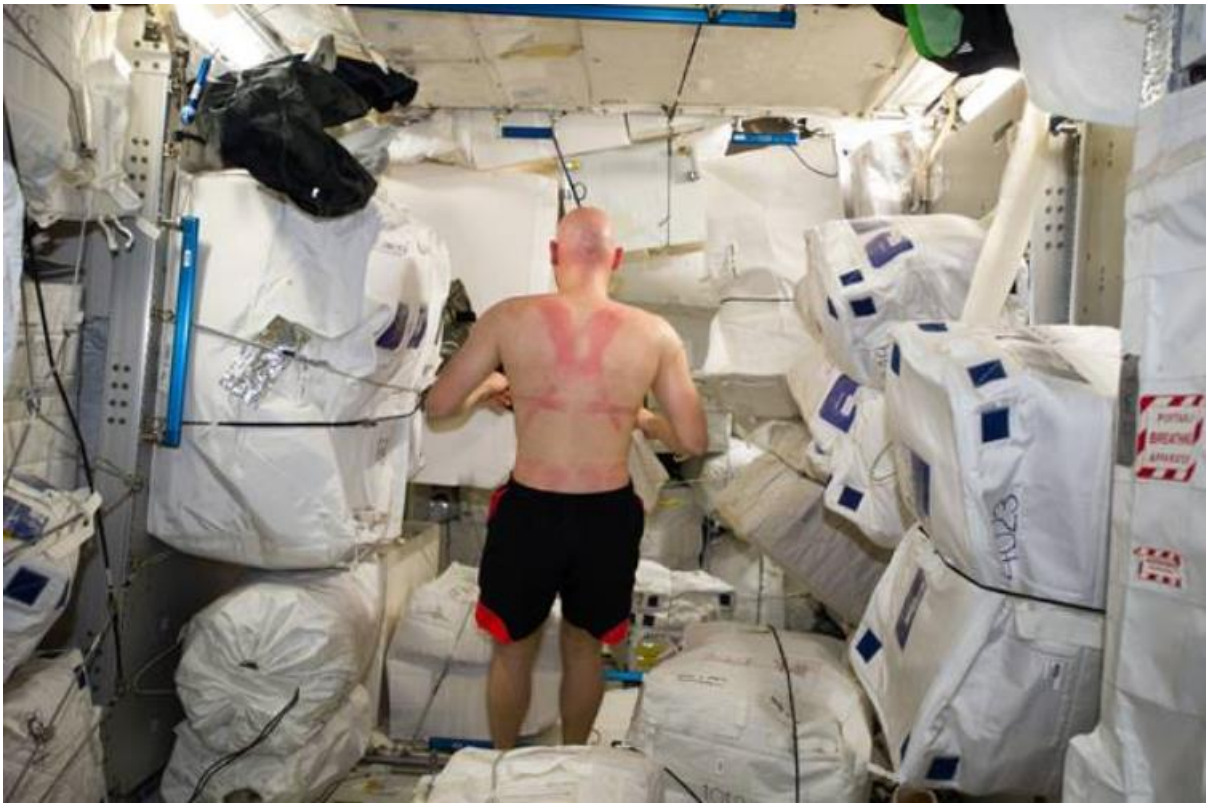

ESA - European Space Agency

Dr. Alexander Gerst
Astronaut

ESTEC – European Space Research and Technology Centre
Keplerlaan 1, NL-2201 AZ Noordwijk, The Netherlands

EAC – European Astronaut Centre
Linder Hoehe, 51147 Cologne, Germany
