# Supplementary material for: Contractile behavior of the gastrocnemius medialis muscle during running in simulated hypogravity
Source: NPJ Microgravity. 2021 Aug 9;7:32. doi: 10.1038/s41526-021-00155-7 (PMC8352871; doi:10.1038/s41526-021-00155-7)
Supplement: Supplementary file 2 — nr-reporting-summary_NPJMGRAV-00567R4 [file 41526_2021_155_MOESM2_ESM.pdf]

nature research

Corresponding author(s): Charlotte Richter

Last updated by author(s): Jun 4, 2021

# Reporting Summary

Nature Research wishes to improve the reproducibility of the work that we publish. This form provides structure for consistency and transparency in reporting. For further information on Nature Research policies, see our Editorial Policies and the Editorial Policy Checklist.

## Statistics

For all statistical analyses, confirm that the following items are present in the figure legend, table legend, main text, or Methods section.

| n/a | Confirmed | |
|---|---|---|
| ☐ | ☒ | The exact sample size (*n*) for each experimental group/condition, given as a discrete number and unit of measurement |
| ☐ | ☒ | A statement on whether measurements were taken from distinct samples or whether the same sample was measured repeatedly |
| ☐ | ☒ | The statistical test(s) used AND whether they are one- or two-sided<br>*Only common tests should be described solely by name; describe more complex techniques in the Methods section.* |
| ☐ | ☒ | A description of all covariates tested |
| ☐ | ☒ | A description of any assumptions or corrections, such as tests of normality and adjustment for multiple comparisons |
| ☐ | ☒ | A full description of the statistical parameters including central tendency (e.g. means) or other basic estimates (e.g. regression coefficient) AND variation (e.g. standard deviation) or associated estimates of uncertainty (e.g. confidence intervals) |
| ☐ | ☒ | For null hypothesis testing, the test statistic (e.g. *F*, *t*, *r*) with confidence intervals, effect sizes, degrees of freedom and *P* value noted<br>*Give P values as exact values whenever suitable.* |
| ☒ | ☐ | For Bayesian analysis, information on the choice of priors and Markov chain Monte Carlo settings |
| ☒ | ☐ | For hierarchical and complex designs, identification of the appropriate level for tests and full reporting of outcomes |
| ☐ | ☒ | Estimates of effect sizes (e.g. Cohen's *d*, Pearson's *r*), indicating how they were calculated |

*Our web collection on statistics for biologists contains articles on many of the points above.*

## Software and code

Policy information about availability of computer code

| | |
|---|---|
| Data collection | Data have been collected using the following hardware/software systems:<br><br>TeleMyo 2400 G2 Telemetry System (Noraxon USA., Inc., Scottsdale, USA), MyoResearch XP software (Master Edition 1.08.16)<br>novel GmbH, loadsol® version 1.4.60, Munich, Germany<br>Prosound α7, ALOKA, Tokyo, Japan<br>Vertical Treadmill Facility (VTF), Arsalis, Glabais, Belgium |
| Data analysis | The obtained data were analyzed using the following software tools:<br><br>A custom-made code to analyze all obtained data was implemented using MATLAB (MathWorks, Inc., Natick, United States), version R2018a. The code is available from the corresponding author upon reasonable request.<br><br>Statistical analysis was performed using the following software:<br>GraphPad Prism, version 7.04<br>G*Power Software, version 3.1.9.4<br><br>A semi-automatic tracking algorithm (UltraTrack Software, version 4.2) was used to quantify GM fascicle lengths and pennation angles. |

For manuscripts utilizing custom algorithms or software that are central to the research but not yet described in published literature, software must be made available to editors and reviewers. We strongly encourage code deposition in a community repository (e.g. GitHub). See the Nature Research guidelines for submitting code & software for further information.

## Data

Policy information about availability of data

All manuscripts must include a data availability statement. This statement should provide the following information, where applicable:

- Accession codes, unique identifiers, or web links for publicly available datasets
- A list of figures that have associated raw data
- A description of any restrictions on data availability

The data that support the findings of this study are available from the corresponding author upon reasonable request.

# Field-specific reporting

Please select the one below that is the best fit for your research. If you are not sure, read the appropriate sections before making your selection.

☒ Life sciences ☐ Behavioural & social sciences ☐ Ecological, evolutionary & environmental sciences

For a reference copy of the document with all sections, see nature.com/documents/nr-reporting-summary-flat.pdf

# Life sciences study design

All studies must disclose on these points even when the disclosure is negative.

| | |
|---|---|
| Sample size | This observational study had a sample size of eight participants. To better address the small sample size, effect sizes were added for all outcome parameters. No sample size calculation was performed prior the study, however, the post-hoc analysis for our primary outcome measure fascicle length revealed a large effect size (dz = 1.7) and high power (1–ß = 0.9). |
| Data exclusions | No data were excluded from the analysis. |
| Replication | All attempts at replication were successful. |
| Randomization | We did not randomize the order of the two loading conditions, due to the overall logistical setup of the experiment. Participants first ran at 1g on a conventional treadmill (control) and thereafter they ran at simulated 0.7g on the vertical treadmill facility. |
| Blinding | Due to the nature of our field of study, it is not possible to blind subjects, because they will immediately notice the difference between running at 1g vs. running at simulated 0.7g (which means running with 30% less body weight). |

# Reporting for specific materials, systems and methods

We require information from authors about some types of materials, experimental systems and methods used in many studies. Here, indicate whether each material, system or method listed is relevant to your study. If you are not sure if a list item applies to your research, read the appropriate section before selecting a response.

## Materials & experimental systems

| n/a | Involved in the study | |
|---|---|---|
| ☒ | ☐ | Antibodies |
| ☒ | ☐ | Eukaryotic cell lines |
| ☒ | ☐ | Palaeontology and archaeology |
| ☒ | ☐ | Animals and other organisms |
| ☐ | ☒ | Human research participants |
| ☒ | ☐ | Clinical data |
| ☒ | ☐ | Dual use research of concern |

## Methods

| n/a | Involved in the study | |
|---|---|---|
| ☒ | ☐ | ChIP-seq |
| ☒ | ☐ | Flow cytometry |
| ☒ | ☐ | MRI-based neuroimaging |

## Human research participants

Policy information about studies involving human research participants

| | |
|---|---|
| Population characteristics | All subjects (males between 20 and 45 years) must have had prior experience running on a treadmill and whose height, and weight characteristics are with the range allowable by the low gravity simulation systems (>170cm and BMI 19-27 kg/m2). Exclusion criteria included cardiovascular, musculoskeletal or neurological diseases and/or surgery within two years prior to participation. |
| Recruitment | Subjects were recruited by King's College London from a pool of males whom had given prior consent to participate in a previous spaceflight related study. Additionally, subjects were also recruited by the Space Medicine Office of the European Space Agency by sending an e-mail to colleagues explaining the purpose and procedures of the study and asking them if they are interested to participate. |
| Ethics oversight | This study received approval from the 'Ärztekammer Nordrhein' Ethical Committee of Düsseldorf, Germany, in accordance with the most recent version of the ethical standards of the 1964 Helsinki declaration. |

Note that full information on the approval of the study protocol must also be provided in the manuscript.
